# Investigating the Thermodynamics and Kinetics of Catechin Pyrolysis for Environmentally Friendly Binders

**DOI:** 10.1021/acsomega.2c07756

**Published:** 2023-03-31

**Authors:** Jakob Kraus, Jens Kortus

**Affiliations:** Institute of Theoretical Physics, TU Bergakademie Freiberg, Leipziger Str. 23, D-09599 Freiberg, Germany

## Abstract

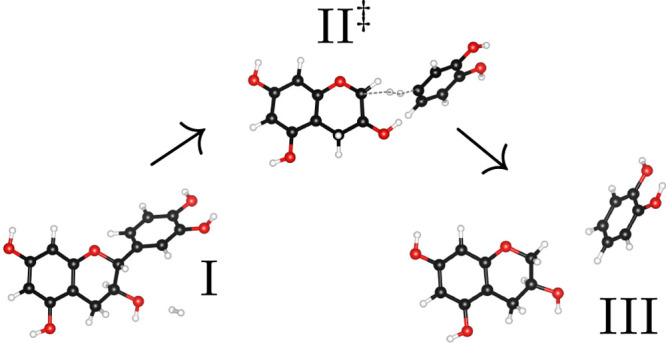

The thermodynamics and kinetics of the pyrolysis of (+)-catechin, a building block of the condensed tannins found in recipes for sustainable binders, are evaluated at the DLPNO-CCSD(T) level and compared to other methods from quantum chemistry. Using the climbing image nudged elastic band method coupled with transition state optimization, minimum energy paths and highest-energy transition states are identified for the first two pyrolysis steps, a catechol split-off with subsequent dehydrogenation. While the catechol split-off path was very smooth, the dehydrogenation featured an additional transition state in the form of an OH group rotation. The combined reaction was judged endothermic in the range of 0 to 1250 K and exergonic at 1000 K and above. It is shown that the catechol split-off is the rate-determining step of the pyrolysis of catechin, which is equivalent to kinetic inhibition at all investigated temperatures.

## 1 Introduction

Condensed tannins are of considerable research interest due to them making up a significant part of the recently developed environmentally friendly binders in carbon-bonded alumina filters for steel melt filtration, namely, in the form of commercially available Quebracho extract, type Indusol ATO.^1−3^

The Quebracho extract, and thus the condensed tannins, have been introduced as binders in combination with lactose with the intent of replacing the currently used binders such as the modified coal tar pitch CarboresP or phenolic resins, i.e., novolaks and resoles.^4^ This replacement is inevitable due to these conventional binders emitting polycyclic aromatic hydrocarbons or phenol at levels which violate the current Registration, Evaluation, Authorisation and Restriction of Chemicals (REACH) regulation of the European Union during binder pyrolysis, the latter of which occurs at the coking step of carbon-bonded filter production.^1,2^ By contrast, the lactose/tannin binders are noncarcinogenic and nontoxic, and their use is both drastically less damaging to the environment and more financially viable in the long run due to REACH conformity, marking them as the sustainable alternative. However, the filters made with environmentally friendly binders suffer from certain thermomechanical disadvantages compared to their state-of-the-art counterparts, including a reduced splitting tensile strength, and the optimization of the binder composition is an ongoing subject of investigation.^1,2^ Since most of the mechanical stability of carbon-bonded filters is dependent on the carbonaceous matrix that is formed between the alumina particles during the coking step, i.e., as a result of binder pyrolysis, the chemical reactions occurring during this phase of filter production and our understanding of them is key to promoting useful advice with respect to favorable binder recipes.

**Figure 1:**
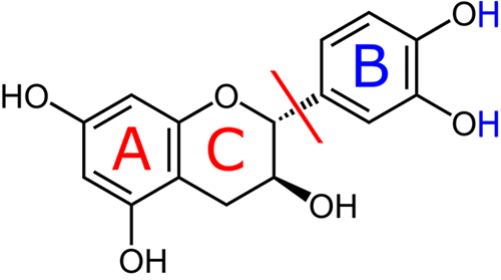
Structural formula of (+)-catechin. The split-off in the first pyrolysis step destroys the C–C bond between the C and the B ring, as marked in red, and the H atoms that are eliminated during the second pyrolysis step are marked in blue.

The chemical reactions under investigation in this study describe the pyrolysis of the central unit of condensed tannins, (+)-catechin, the most commonly encountered catechin stereoisomer. Here, we limit ourselves to the first two steps of catechin pyrolysis. In the combined pyrolysis/gas chromatography/mass spectrometry study by Galletti et al.,^5^ catechol was revealed as the most prominent pyrolysis product of catechin. Catechol was also measured after the pyrolysis of methylated catechin (Galletti et al.^6^) and the silylated version of the condensed tannin procyanidin C1 (Mattonai et al.^7^), albeit to a lesser degree. Furthermore, both Thomas et al.^8^ and Lomnicki et al.^9^ discussed the possibility of *o*-benzoquinone forming as an intermediate during catechol pyrolysis. Thus, the two reactions
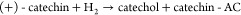
1and
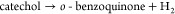
2i.e., a catechol split-off followed by a dehydrogenation of the emerging catechol, are proposed for this study. The rest of the catechin after the split-off consists of the A and C rings of (+)-catechin, as presented in Figure 1, which is why we refer to this molecule as catechin-AC. Moreover, the presence of H_2_ in Equation 1 is motivated by the presence of both a petroleum coke bed and the additive carbon black during the coking of the carbon-bonded filters in practice.^1−3^ In addition to the H_2_ obtained from volatile hydrocarbons leaving coke and carbon black during pyrolysis, the second reaction generates additional H_2_ to further react with (+)-catechin in the first reaction.

In order to study the chemical reactions presented in Equation 1 and Equation 2, the climbing image nudged elastic band method (CI-NEB^10−13^) is employed in this study. CI-NEB is a state-of-the-art approach of identifying minimum energy paths on the nuclear potential energy surface, connecting the local minima known as reactants and products of a given chemical reaction. Combined with further transition state optimization, CI-NEB allows access to the highest-energy transition states which are related to the rearrangement of chemical bonds during the reaction. From these transition states, data covering the kinetics of this reaction can be extracted, in addition to the thermodynamic information gained from the reactant/product comparisons.

In this work, CI-NEB is applied in combination with Kohn–Sham density functional theory (DFT^14,15^) to yield minimum energy paths and transition states, whereas the final energetics are evaluated using coupled cluster theory with single and double excitations as well as perturbative triple excitations (CCSD(T)^16−18^), along with a variety of other wave function-based ansatzes and DFT methods. A previous study by Kraus et al. on gallic acid, the building block of another class of tannins, the gallotannins, has already demonstrated the capability of CI-NEB calculations to gain insight into tannin pyrolysis, as it was able to trace the loss of carbon during this particular pyrolysis reaction,^19^ a phenomenon which is generally linked to the cold crushing strength of the resulting filter.^1^ To the best of our knowledge, the minimum energy paths corresponding to the two catechin pyrolysis reactions described above have not been investigated so far.

## 2 Computational Details

The quantum chemical calculations in this study were executed using the ORCA code,^20,21^ version 4.2.1. For a deep dive look at the implementation of the CI-NEB functionality and the subsequent transition state optimization in ORCA, we recommend the work by Ásgeirsson et al. on the subject.^13^

### 2.1 Geometry Optimization and Vibrational Analysis

As a first step, the database structures documented in Table 1, which correspond to most of the molecules taking part in the two investigated catechin pyrolysis reactions, were subjected to geometry optimization. An exception was made for the catechin-AC molecule, which was generated by hand using the ASE(22) program and subjected to the very same optimization.

**Table 1:** Source of Initial Nuclear Geometries for Molecules Partaking in Catechin Pyrolysis Reactions^a^

Molecule	Database	Identifier
(+)-catechin	PubChem	PubChem CID 9064
H_2_	ChemSpider	ChemSpider ID 762
catechol	PubChem	PubChem CID 289
*o*-benzoquinone	PubChem	PubChem CID 11421

a The databases PubChem(23,24) and ChemSpider(25) were consulted.

The following parameters were employed in the geometry optimizations:

- exchange-correlation functional: PBE^26^
- basis set: pc-3^27,28^ (valence quadruple-ζ)
- integration grid: ORCA grid level 7, i.e., (45,770) for H atoms and (50,770) for C and O atoms; unpruned
- SCF energy tolerance: 10^–8^ a.u.
- optimizer: BFGS^29−32^
- maximum atomic force: 10^–4^ a.u.
- RMS atomic force: 3 × 10^–5^ a.u.
- maximum energy change between geometries: 10^–6^ a.u.

After the optimizations were completed, PBE vibrational frequencies were calculated numerically and checked for imaginary frequencies in order to confirm the status of the optimized structures as local minima on the nuclear potential energy surface. As none were found, these optimized structures were used as starting points for joint geometry optimizations for the reactants and products of the two examined catechin pyrolysis reactions. This meant optimizations for the pairs (+)-catechin/H_2_, catechol/catechin-AC, and *o*-benzoquinone/H_2_. The joint optimizations were performed using parameters identical to the ones listed above, and again, numerical vibrational analysis was performed after convergence. When it was confirmed that the joint reactants and products were local minima as well, the CI-NEB calculations were started.

### 2.2 CI-NEB and Transition State Optimization

The CI-NEB calculations in this study were performed mostly using the same parameters that were previously employed for the geometry optimizations. However, the pc-3 basis set was exchanged for the valence triple-ζ pc-2 basis set for efficiency reasons. In addition, the first CI-NEB phase, the identification of the CI, saw the following calculation parameters:

- initial path generation: IDPP^33^
- end points: fixed
- optimizer: L-BFGS^34−38^
- number of images: 30 + 2 (reactants, products)
- springs: energy-weighted (0.01 a.u. to 0.1 a.u.)
- maximum perpendicular atomic force: 0.02 a.u.

When the maximum perpendicular atomic force dropped below the mentioned threshold, the CI was identified. Then, the CI and the regular images were optimized with differing convergence criteria in the second phase of the CI-NEB calculations, still using the L-BFGS optimizer:

- maximum atomic force (CI): 0.002 a.u.
- RMS atomic force (CI): 0.001 a.u.
- maximum perpendicular atomic force (regular images): 0.02 a.u.
- RMS perpendicular atomic force (regular images): 0.01 a.u.

Following the success of the CI-NEB calculation, the converged CI was subjected to a transition state optimization using an eigenvector-following method. This optimization employed the following parameters:

- maximum atomic force: 3 × 10^–4^ a.u.
- RMS atomic force: 10^–4^ a.u.
- maximum energy change between geometries: 5 × 10^–6^ a.u.

As with the simpler optimized structures before, a vibrational analysis was performed on the optimized transition states. Here, the transition states each exhibited a single imaginary frequency, cementing their status as first-order saddle points on the potential energy surface.

### 2.3 Thermodynamics and Kinetics

The thermodynamic quantities of interest, namely, the standard enthalpy of reaction Δ_r_*H*° and the standard Gibbs energy of reaction Δ_r_*G*°, were determined on the optimized individual reactant and product molecules for the temperatures 0 K, 273 K, 298 K, 500 K, 750 K, 1000 K, and 1250 K, allowing for comparisons to the gallic acid data reported in Kraus et al.^19^ In order to gather information on kinetics for the aforementioned temperatures, standard Gibbs energies of activation Δ^‡^*G*° and reaction rate constants *k* were calculated too. For the standard Gibbs energies of activation, the optimized highest-energy transition states from the CI-NEB calculations were evaluated with respect to the individual reactant molecules. On the other hand, *k* was computed according to^39^
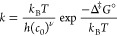
3with *c*_0_ = 1 M as the standard concentration as well as ν = 0 for unimolecular reactions and ν = 1 for bimolecular reactions. In all cases, the ideal gas approximation as implemented in ASE was employed, setting the pressure to *p* = 1 bar to account for the presumed standard state of the gases.^40^

Enthalpies and Gibbs energies were calculated from pc-3 PBE vibrational frequencies and from total energies originating from seven different quantum chemical approaches. Apart from PBE, the DFT functionals SCAN^41^ and B3LYP^42−46^ were chosen to represent the meta-GGA and hybrid functional classes, respectively. As such, the kinetic energy density is used in the construction of the SCAN functional and a portion (20%) of exact exchange is employed in the B3LYP functional, in addition to the dependence on both the local density and the local density gradient that is already present in the PBE functional. Compared to the GGA functional PBE, which occupies the second rung on the so-called Jacob’s ladder of DFT,^47^ SCAN and B3LYP are higher-level approximations to the unknown exact exchange-correlation functional, residing on the third and fourth rung of the ladder, respectively. Moreover, Hartree–Fock (HF) theory,^48,49^ second-order Møller–Plesset perturbation (MP2) theory,^50^ coupled cluster theory with single and double excitations (CCSD),^16−18^ and finally CCSD(T) were applied, the latter of which is used as reference throughout this work. In contrast to the DFT methods PBE, SCAN, and B3LYP, these four methods aim for approximating the exact wave function of the interacting many-body system. The HF wave function is the Slater determinant with the lowest total energy for a given system, and the post-HF methods MP2, CCSD, and CCSD(T) build on HF theory by introducing charge correlation, which is missing in HF. Whereas MP2 uses second-order Rayleigh–Schrödinger perturbation theory to account for the correlation energy, coupled cluster theory, here represented by CCSD and CCSD(T), does so by applying the cluster operator, which causes electronic excitations, to the HF Slater determinant following an exponential approach. For CCSD, the cluster operator is truncated after the single and double excitation operators, and for CCSD(T), triple excitations are additionally included via perturbation theory. In this study, CCSD(T) was chosen as reference due to its reputation as the “gold standard” of quantum chemistry,^51^ offering high accuracy with respect to experiment for a computational cost that is often affordable. For all seven quantum chemical methods, the total energies were calculated using the pc-3 basis set and an SCF energy tolerance of 10^–8^ a.u.; for the DFT methods, the integration mesh was identical to the one described in Subsection 2.1 for the geometry optimizations.

All post-HF methods employed the frozen core approximation, neglecting excitations from the two inner electrons of the C and O atoms. Notably, the CCSD and CCSD(T) calculations were made possible due to the linearly scaling domain based local pair natural orbital (DLPNO) procedure.^52−54^ When applying the DLPNO scheme, the default DLPNO truncation thresholds in ORCA were employed, which corresponds to the NormalPNO keyword. For DLPNO-CCSD(T), the so-called semicanonical approximation, also known as DLPNO-CCSD(T0), was used for the perturbative triple excitations. In the context of this manuscript, all mentions of DLPNO-CCSD(T) do in fact refer to DLPNO-CCSD(T0), including in the tables and the Supporting Information.

## 3 Results and Discussion

### 3.1 CI-NEB Minimum Energy Paths

**Figure 2:**
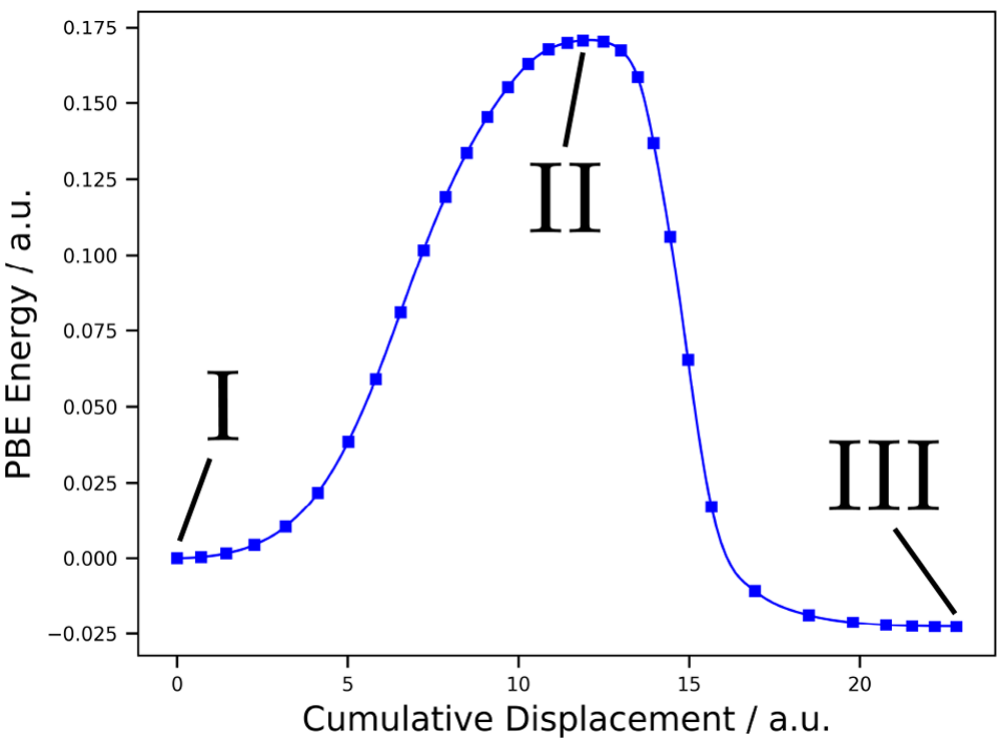
Converged CI-NEB minimum energy path of the first reaction (+)-catechin + H_2_ → catechol + catechin-AC. The quantity on the *x* axis, the cumulative displacement between subsequent images, is considered the reaction coordinate. The dots denote the pc-2 PBE energies of the images relative to the first image, and the line is an interpolating spline. Code for selected images: I, (+)-catechin/H_2_; II, converged CI; III, catechol/catechin-AC. The figure was created with the help of a Python script by Ásgeirsson.^55^

The converged CI-NEB minimum energy path for the first catechin pyrolysis step, the catechol split-off (+)-catechin + H_2_ → catechol + catechin-AC, is illustrated in Figure 2. The path is given by showing the pc-2 PBE total energy relative to the first image, i.e., the starting image I - (+)-catechin/H_2_, as a function of the cumulative displacement between subsequent images. This displacement can be identified with the reaction coordinate of transition-state theory.

Apart from the starting image I - (+)-catechin/H_2_ (image 0) and the final image III - catechol/catechin-AC, the minimum energy path in Figure 2 sports a global maximum at image 17, designated by II - the converged CI, which was then subjected to transition state optimization. This minimum energy path is smooth and shows a multitude of images close in energy to the CI, which makes the global maximum look rather broad. Moreover, one can clearly observe that the reaction is predicted to be slightly exothermic by PBE at 0 K under the *caveat* of using pc-2 and neglecting any zero-point vibrational energy contributions.

**Figure 3:**
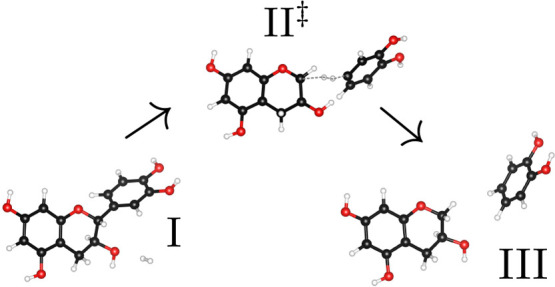
Selected images of the minimum energy path for the first reaction (+)-catechin + H_2_ → catechol + catechin-AC. II^‡^ represents the optimized transition state for which II acted as a starting point. Color code: white, H atoms; black, C atoms; red, O atoms. The solid lines represent fully formed bonds, and the dotted lines represent bonds in the process of being broken or formed.

The three special images I, II^‡^ - the optimized transition state, and III are presented in Figure 3, showing the mechanistic side of this first reaction. Compared to the converged CI (designated by II), II^‡^ is lower in pc-2 PBE energy by 12.4 kcal mol^–1^, a deviation that is mainly rooted in a different orientation of the catechol moiety with respect to the catechin-AC rest of the molecule and a rotation of the OH group close to the center of reaction. We see that the H_2_ molecule approaches the C–C bond connecting the C and B rings of (+)-catechin between images I and II^‡^ and that this C–C bond is already broken before the actual transition state is reached. The transition state II^‡^ itself shows the simultaneous break of the H–H bond and the forming of two new C–H bonds, one at the C ring, i.e., the catechin-AC rest, and one at the B ring, i.e., the catechol moiety. Afterward, the two product molecules catechin-AC and catechol separate, leading to configuration III.

**Figure 4:**
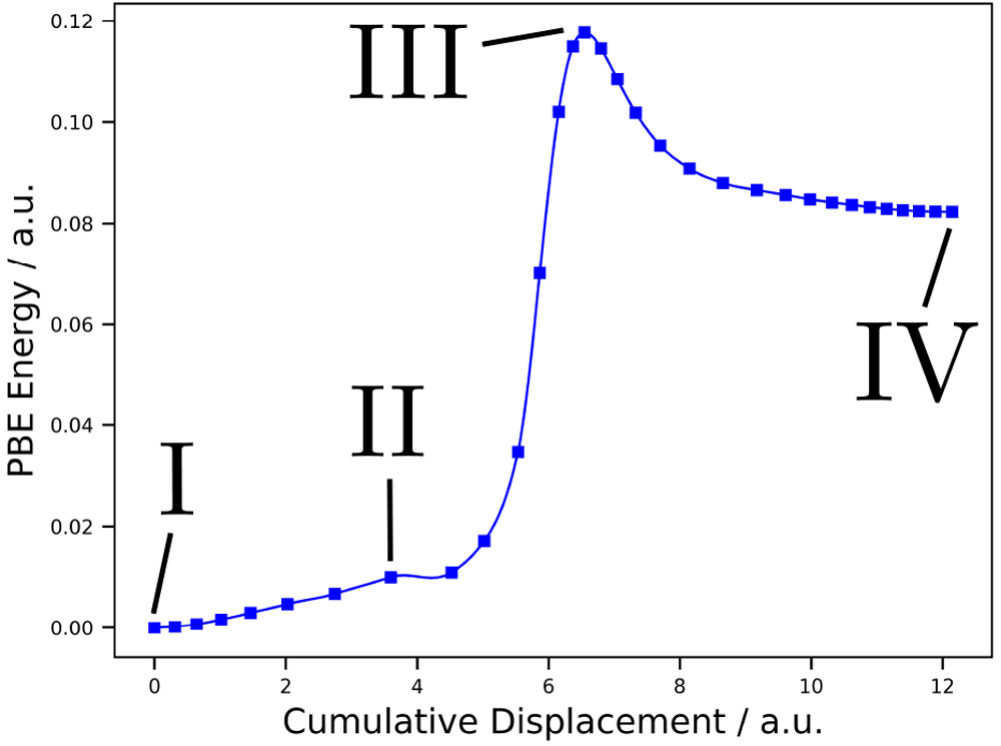
Converged CI-NEB minimum energy path of the second reaction catechol → *o*-benzoquinone + H_2_. Code for selected images: I, catechol; II, rotational transition state; III, converged CI; IV, *o*-benzoquinone/H_2_. The figure was created with the help of a Python script by Ásgeirsson.

The converged CI-NEB minimum energy path for the second catechin pyrolysis step, the catechol dehydrogenation catechol → *o*-benzoquinone + H_2_, is given in Figure 4. This minimum energy path sports two images of interest apart from the starting image I - catechol (image 0) and the final image IV - *o*-benzoquinone/H_2_. Both of these images, which are designated by II and III, respectively, are maxima and therefore transition state candidates. The first one, image 7, is a local maximum, and vibrational analysis confirmed a single imaginary frequency, marking it as a transition state. The second one, image 14, is the converged CI, and thus the global maximum to be optimized further. Compared to Figure 2, the CI peak in the minimum energy path is sharper, and the existence of an additional lower-energy transition state before the highest-energy one is another feature not found in the first reaction. Furthermore, this reaction is calculated to be distinctly endothermic, which is another contrast. In total, the minimum energy path of the second reaction greatly resembles the path for the second reaction of gallic acid pyrolysis as described in Kraus et al.,^19^ which is expected given the fact that both reactions are dehydrogenations and catechol and pyrogallol only differ by the additional OH group of the latter.

**Figure 5:**
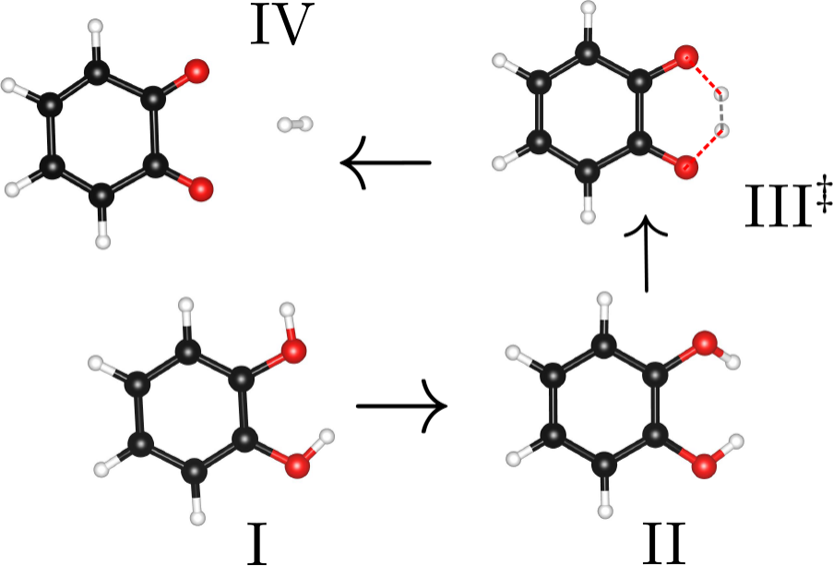
Selected images of the minimum energy path for the second reaction catechol → *o*-benzoquinone + H_2_. III^‡^ represents the optimized transition state for which III acted as a starting point. Color code: white, H atoms; black, C atoms; red, O atoms. The solid lines represent fully formed bonds, and the dotted lines represent bonds in the process of being broken or formed.

Figure 5 displays the four most important images for the second reaction, including the optimized transition state III^‡^. This transition state possesses a pc-2 PBE energy that is 0.1 kcal mol^–1^ lower than that of III, the converged CI. This minimal energy change corresponds to no significant visually discernible molecular rearrangement between III and III^‡^. From Figure 5, it is apparent that II is in fact a rotational transition state, as one of the two catechol OH groups undergoes a rotation before the highest-energy transition state III^‡^ is formed. This highest-energy transition state includes the break of the two O−H bonds, while the H–H bond of H_2_ is formed at the same time. The nature of both transition states is, again, in agreement with the results previously evaluated for gallic acid pyrolysis.^19^ Finally, IV shows *o*-benzoquinone and H_2_ as fully disconnected molecular structures.

### 3.2 Thermodynamics

In Table 2, the main thermodynamic and kinetic data as calculated in this study are reported. This includes DLPNO-CCSD(T) standard enthalpies of reaction Δ_r_*H*°_DLPNO-CCSD(T)_, standard Gibbs energies of reaction Δ_r_*G*°_DLPNO-CCSD(T)_, and standard Gibbs energies of activation Δ^‡^*G*°_DLPNO-CCSD(T)_ at the pc-3 level for the first pyrolysis reaction (+)-catechin + H_2_ → catechol + catechin-AC and the second pyrolysis reaction catechol → *o*-benzoquinone + H_2_ as a function of temperature. In addition, Δ_r_*H*°_DLPNO-CCSD(T)_ and Δ_r_*G*°_DLPNO-CCSD(T)_ are given for the combined reaction (+)-catechin → catechin-AC + *o*-benzoquinone.

**Table 2:** pc-3 DLPNO-CCSD(T) Standard Enthalpies of Reaction Δ_r_*H*°_DLPNO-CCSD(T)_ and Standard Gibbs Energies of Reaction Δ_r_*G*°_DLPNO-CCSD(T)_ for the First Reaction (+)-Catechin + H_2_ → Catechol + Catechin-AC, the Second Reaction Catechol → *o*-Benzoquinone + H_2_, and the Combined Reaction (+)-Catechin → Catechin-AC + *o*-Benzoquinone as a Function of Temperature *T*^a^

	*T*						
	0	273	298	500	750	1000	1250
(+)-catechin + H_2_ → catechol + catechin-AC							
Δ_r_*H*°_DLPNO-CCSD(T)_	–7.5	–9.1	–9.3	–10.6	–11.8	–12.7	–13.5
Δ_r_*G*°_DLPNO-CCSD(T)_	–7.5	–12.6	–12.9	–15.0	–16.9	–18.4	–19.7
Δ^‡^*G*°_DLPNO-CCSD(T)_	115.0	119.9	120.5	125.1	130.8	136.5	142.1
catechol → *o*-benzoquinone + H_2_							
Δ_r_*H*°_DLPNO-CCSD(T)_	43.6	45.3	45.4	46.0	46.2	46.2	45.9
Δ_r_*G*°_DLPNO-CCSD(T)_	43.6	37.1	36.3	30.0	21.9	13.8	5.8
Δ^‡^*G*°_DLPNO-CCSD(T)_	85.8	86.0	86.0	86.3	86.6	87.0	87.3
(+)-catechin → catechin-AC + *o*-benzoquinone							
Δ_r_*H*°_DLPNO-CCSD(T)_	36.1	36.2	36.1	35.4	34.4	33.5	32.4
Δ_r_*G*°_DLPNO-CCSD(T)_	36.1	24.5	23.4	15.0	5.0	–4.6	–13.9

a For the first and second reactions, the standard Gibbs energies of activation Δ^‡^*G*°_DLPNO-CCSD(T)_ are given as well. Δ_r_*H*°_DLPNO-CCSD(T)_, Δ_r_*G*°_DLPNO-CCSD(T)_, and Δ^‡^*G*°_DLPNO-CCSD(T)_ are given in kcal mol^–1^, *T* is given in K.

Moreover, Table 3 displays how much the remaining applied approaches deviate from the DLPNO-CCSD(T) zero-temperature standard enthalpy of reaction Δ_r_*H*°(0 K)_DLPNO-CCSD(T)_ and the zero-temperature standard Gibbs energy of activation Δ^‡^*G*°(0 K)_DLPNO-CCSD(T)_. Due to the use of PBE vibrational frequencies in the construction of all the enthalpies and Gibbs energies in this study, these listed differences are the only data necessary to reconstruct Table 2 for PBE, SCAN, B3LYP, HF, MP2, and DLPNO-CCSD.

**Table 3:** Deviations of the pc-3 Zero-Temperature Standard Enthalpies of Reaction Δ_r_*H*°(0 K) from the Respective DLPNO-CCSD(T) Results Δ_r_*H*°(0 K)_DLPNO-CCSD(T)_ for the First Reaction (+)-Catechin + H_2_ → Catechol + Catechin-AC, the Second Reaction Catechol → *o*-Benzoquinone + H_2_, and the Combined Reaction (+)-Catechin → Catechin-AC + *o*-Benzoquinone as a Function of the Calculation Method^a^

	PBE	SCAN	B3LYP	HF	MP2	DLPNO-CCSD
(+)-catechin + H_2_ → catechol + catechin-AC						
Δ_r_*H*°(0 K) – Δ_r_*H*°(0 K)_DLPNO-CCSD(T)_	–2.9	–0.9	–6.0	–9.7	3.1	–1.1
Δ^‡^*G*°(0 K) – Δ^‡^*G*°(0 K)_DLPNO-CCSD(T)_	–24.5	–16.8	–13.4	16.8	3.4	5.8
catechol → *o*-benzoquinone + H_2_						
Δ_r_*H*°(0 K) – Δ_r_*H*°(0 K)_DLPNO-CCSD(T)_	0.1	3.8	–2.4	–3.2	5.6	0.5
Δ^‡^*G*°(0 K) – Δ^‡^*G*°(0 K)_DLPNO-CCSD(T)_	–18.6	–12.4	–9.2	24.4	–2.4	5.9
(+)-catechin → catechin-AC + *o*-benzoquinone						
Δ_r_*H*°(0 K) – Δ_r_*H*°(0 K)_DLPNO-CCSD(T)_	–2.8	2.9	–8.4	–12.9	8.7	–0.6

a For the first and second reactions, the deviations of the zero-temperature standard Gibbs energies of activation Δ^‡^*G*°(0 K) from the respective DLPNO-CCSD(T) results Δ^‡^*G*°(0 K)_DLPNO-CCSD(T)_ are given as well. All deviations are given in kcal mol^–1^.

Inspecting the data in Table 2, the combined reaction for the pyrolysis of (+)-catechin is predicted to be endothermic for the full temperature range up to 1250 K, as the consistently endothermic second reaction always overcompensates the consistently exothermic first reaction. The temperature dependence of Δ_r_*H*°_DLPNO-CCSD(T)_ for the combined reaction is rather limited. The values mostly decrease with increasing temperature, except in the very low temperature range, with a maximum difference of 3.8 kcal mol^–1^ between the maximum at 273 K and the minimum at 1250 K. All of these trends are very similar to the results reported by Kraus et al. for gallic acid pyrolysis,^19^ which featured an exothermic first reaction, a dominant endothermic second reaction, and Δ_r_*H*°_DLPNO-CCSD(T)_ data in the range of 36.2 kcal mol^–1^ to 38.5 kcal mol^–1^ for the combined reaction. This is not surprising considering the closeness in the minimum energy paths for the respective second reactions, the dehydrogenations, as discussed earlier.

An experimental value was available for Δ_r_*H*°(298 K) of the second reaction, enabling comparisons to DLPNO-CCSD(T) and the other applied approaches. Taking the recommended value for the standard enthalpy of formation Δ_f_*H*°(298 K) of *o*-benzoquinone (−21.0 kcal mol^–1^) from Fattahi et al.^56^ and the corresponding value of catechol (−65.7 kcal mol^–1^) from Sabbah et al.,^57^ we arrive at an experimental result of Δ_r_*H*°(298 K) = 44.7 kcal mol^–1^. This is in excellent agreement with the DLPNO-CCSD(T) value of 45.4 kcal mol^–1^, as this deviation of 0.7 kcal mol^–1^ is within the chemical accuracy of 1.0 kcal mol^–1^. According to Table 3, the PBE results also fulfill the criterion of chemical accuracy and the DLPNO-CCSD ones come very close, with deviations from experiment of 0.8 kcal mol^–1^ and 1.2 kcal mol^–1^, respectively. Whereas B3LYP and HF slightly underestimate the experimental value, with deviations of −1.7 kcal mol^–1^ and −2.5 kcal mol^–1^, respectively, SCAN (deviation: 4.5 kcal mol^–1^) and MP2 (deviation: 6.3 kcal mol^–1^) deliver results which are too high. Considering the experimental uncertainties of 3.1 kcal mol^–1^ for *o*-benzoquinone^56^ and 0.3 kcal mol^–1^ for catechol,^57^ the propagated uncertainty for Δ_r_*H*°(298 K) = 44.7 kcal mol^–1^ is equal to 3.4 kcal mol^–1^. This means that the PBE, DLPNO-CCSD, B3LYP, and HF results are actually within chemical accuracy if uncertainties are taken into consideration. For the first pyrolysis reaction, no sufficient experimental data were available for comparison. In general, all non-DLPNO-CCSD(T) methods share the prediction of an endothermic combined reaction for the full temperature range.

As for Δ_r_*G*°_DLPNO-CCSD(T)_, the combined reaction is estimated to alter its behavior from endergonic to exergonic between 750 and 1000 K, becoming increasingly exergonic with rising temperature because of both the consistently exergonic first reaction becoming more exergonic and the consistently endergonic second reaction becoming less endergonic. Again, these findings are analogous to the findings by Kraus et al., with the only exception being that the switch from endergonic to exergonic was predicted at slightly lower temperatures for gallic acid. This means that, both in terms of the enthalpies of reaction (the generation of products of endothermic reactions is favored as the temperature rises due to Le Chatelier’s principle) and in terms of the Gibbs energies of reaction (reaction becomes exergonic, i.e. it can happen spontaneously, as the temperature rises), the combined pyrolysis reaction of catechin profits from the presence of high temperatures. In contrast to Δ_r_*H*°_DLPNO-CCSD(T)_, the Gibbs energy of reaction exhibits a pronounced temperature dependency, sporting a decrease of 50.0 kcal mol^–1^ when the data for 0 and 1250 K are compared. The Gibbs energies of reaction are always lower than the enthalpies of reaction. This observation can be resolved by remembering that the second reaction features more products than reactants, and thus possesses a positive entropy of reaction, lowering the Gibbs energy in the process; this effect is not countered by the first reaction due to an equal number of reactants and products.

Because of significant deviations from the Δ_r_*H*°(0 K)_DLPNO-CCSD(T)_ values, B3LYP and HF already foretell an exergonic combined reaction at 750 K, whereas the MP2 results only change to exergonic for 1250 K. All in all, the closest agreement with DLPNO-CCSD(T) for the combined reaction is achieved by the related method of DLPNO-CCSD, with a deviation of −0.6 kcal mol^–1^, i.e., below chemical accuracy, followed by PBE (deviation: −2.8 kcal mol^–1^) and SCAN (deviation: 2.9 kcal mol^–1^). Unsurprisingly, the least agreement is achieved by HF, with a deviation of −12.9 kcal mol^–1^, as this is the only approach that does not include any electron charge correlation whatsoever.

### 3.3 Kinetics

The DLPNO-CCSD(T) standard Gibbs energies of activation Δ^‡^*G*°_DLPNO-CCSD(T)_ are presented in Table 2, and the respective reaction rate constants *k*_DLPNO-CCSD(T)_ are presented in Tables S1 (for the first reaction) and S2 (for the second reaction). A comparison of the Gibbs energies of activation makes it clear that the first reaction step, the catechol split-off, is the rate-determining step of catechin pyrolysis. The Δ^‡^*G*°_DLPNO-CCSD(T)_ values for the first reaction (115.0 kcal mol^–1^ to 142.1 kcal mol^–1^) are significantly higher than those of the second reaction throughout, with a difference of 29.2 kcal mol^–1^ at 0 K increasing with temperature to a difference of 54.8 kcal mol^–1^ at 1250 K. While both barrier heights grow with temperature, that of the first reaction does so far more drastically, exhibiting an increase of 27.1 kcal mol^–1^ across the full temperature range compared to the tiny 1.5 kcal mol^–1^ growth for the second reaction. Owing to the similarity of the minimum energy paths, the Δ^‡^*G*°_DLPNO-CCSD(T)_ results for the dehydrogenation are in close agreement with the pyrogallol dehydrogenation data reported in Kraus et al.,^19^ deviating by less than 2.0 kcal mol^–1^ across all temperatures. Moreover, the best agreement with DLPNO-CCSD(T) is achieved by MP2, with Δ^‡^*G*° results that differ by less than 3.5 kcal mol^–1^ for both reactions.

The difference between the Δ^‡^*G*°_DLPNO-CCSD(T)_ results for the two reactions and the mentioned agreement with earlier work is reflected in the respective *k*_DLPNO-CCSD(T)_ results as well. In fact, the numerical values for the first reaction are lower than those of the second reaction by at least 10 orders of magnitude, although the constants themselves should not be compared directly due to the difference in unit. Since the first reaction is bimolecular while the second reaction is unimolecular, at least one of the two reactants in the first reaction must sport a concentration of at least 10^5^*c*_ref_ to make the corresponding reaction rates of the two reactions roughly equal, with *c*_ref_ ≤ 1 M being the concentration of the reactant in the second reaction, i.e., catechol. The assumption of the catechol concentration being lower than or equal to 1 M is justified by the setup of the binder coking process, as there is no catechol present before the onset of pyrolysis. Moreover, the unimolecular reaction rate constants are of the same order of magnitude as the previously reported unimolecular pyrogallol dehydrogenation reaction rate constants at 500 K and above, and just 1 order of magnitude off for lower temperatures.^19^ Although all reaction rate constants continuously and enormously increase with temperature as expected according to Equation 3, the value for the first reaction is only 3.73 × 10^–12^ M^–1^ s^–1^ at 1250 K, which is a very small value. Test calculations have revealed that the reaction rate constant for the first reaction becomes larger than 10^–10^ M^–1^ s^–1^ for temperatures of 1500 K and above, and larger than 1 M^–1^ s^–1^ for temperatures of 3000 K and above. Consequently, the pyrolysis of (+)-catechin as studied in this paper is considered thermodynamically favorable starting at 1000 K; however, it is kinetically inhibited in the total primarily explored temperature range up to and including 1250 K.

## 4 Conclusions

In this work, we have provided minimum energy paths and thermodynamic as well as kinetic data on the first two reactions of (+)-catechin pyrolysis, i.e., a catechol split-off followed by a dehydrogenation, using the DFT functional PBE/pc-2 for the CI-NEB calculations and DLPNO-CCSD(T)/pc-3 as reference for the evaluation of energies. For both reaction steps, the minimum energy paths were marked by highest-energy transition states related to the rearrangement of chemical bonds. The second reaction also featured a rotational transition state, in accordance with a similar reaction found in gallic acid pyrolysis.^19^

In terms of enthalpies, the combined reaction was judged endothermic from 0 to 1250 K, yielding Δ_r_*H*°_DLPNO-CCSD(T)_ in the range of 32.4 kcal mol^–1^ to 36.2 kcal mol^–1^. Moreover, this combined reaction was predicted to change from endergonic to exergonic between 750 and 1000 K, with the Δ_r_*G*°_DLPNO-CCSD(T)_ value at −13.9 kcal mol^–1^ for a temperature of 1250 K. Thus, an increase of temperature significantly improves the thermodynamic stability of the pyrolysis products compared to the reactants. Among the other tested methods, DLPNO-CCSD achieved the best agreement with DLPNO-CCSD(T), exhibiting a deviation of only −0.6 kcal mol^–1^. Furthermore, an experimental value for Δ_r_*H*°(298 K) was found for the dehydrogenation, and the DLPNO-CCSD(T) result deviated by only 0.7 kcal mol^–1^, which is within chemical accuracy.

On the kinetic side of things, the catechol split-off was clearly identified as the rate-determining step of catechin pyrolysis with a high Δ^‡^*G*°_DLPNO-CCSD(T)_ result in the range of 115.0 kcal mol^–1^ to 142.1 kcal mol^–1^. In fact, based on the calculated reaction rate constants *k*_DLPNO-CCSD(T)_, the combined reaction is not expected to take place in a significant way up to and including temperatures of 1250 K due to kinetic inhibition. As a consequence, we believe that the role of catalytically active substances which were not incorporated in this study and which have been present during the coking of carbon-bonded filters in the past, such as graphite and SiO_2_,^1−4^ the latter of which was reported to speed up certain reactions of condensed tannins,^4^ should be explored in future studies covering sustainable binders based on lactose/tannin. Moreover, as mentioned in the introduction, we have limited ourselves to only two possible reactions related to the pyrolysis of (+)-catechin in this study. Other reactions which might also play a role in the pyrolysis of this molecule include, e.g., hydrogenations of the aromatic A and B rings or O–H bond scissions leading to the formation of radical species. Thus, recovering the minimum energy paths of these competing reaction channels might shed more light on the phenomenon of catechin pyrolysis in future investigations.

For reference, .XYZ files containing the nuclear coordinates of all images along the minimum energy paths presented in Figure 2 and Figure 4 as well as .XYZ files containing the nuclear coordinates of the selected images presented in Figure 3 and Figure 5 are available at GitLab (https://gitlab.com/jakobkraus/publication_data). In this repository, .GIF files showing the reactions can be found as well.

## Supporting Information Available

Tabulated reaction rate constants for reactions 1 and 2 (PDF)
