# Supplementary material for: Investigating the Thermodynamics and Kinetics of Catechin Pyrolysis for Environmentally Friendly Binders
Source: ACS Omega. 2023 Mar 31;8(14):12693–701. doi: 10.1021/acsomega.2c07756 (PMC10099430; doi:10.1021/acsomega.2c07756)
Supplement: Supplementary file 1 — ao2c07756_si_001.pdf [file ao2c07756_si_001.pdf]

Investigating the thermodynamics and kinetics of catechin pyrolysis for environmentally friendly binders - Supporting Information -

Jakob Kraus[a*] and Jens Kortus[a]

[a] TU Bergakademie Freiberg, Institute of Theoretical Physics,

Leipziger Str. 23, D-09599 Freiberg, Germany

[*] jakob.kraus@physik.tu-freiberg.de, [†] jens.kortus@physik.tu-freiberg.de

Table S1: Bimolecular reaction rate constants k for the first reaction (+)-catechin + H_2 $\rightarrow$ catechol + catechin-AC as a function of temperature T and calculation method. k is given in $M^{-1}\,s^{-1}$, T is given in K.

T	PBE	SCAN	B3LYP	HF	MP2	DLPNO-CCSD	DLPNO-CCSD(T)
0	0.00E+00	0.00E+00	0.00E+00	0.00E+00	0.00E+00	0.00E+00	0.00E+00
273	2.01E-64	1.66E-70	3.15E-73	1.75E-97	1.12E-86	1.34E-88	5.90E-84
298	2.45E-58	5.52E-64	1.77E-66	1.26E-88	8.47E-79	1.47E-80	2.64E-76
500	1.11E-31	4.80E-35	1.57E-36	9.88E-50	7.11E-44	6.35E-45	2.18E-42
750	1.65E-18	9.44E-21	9.64E-22	1.53E-30	1.23E-26	2.45E-27	1.20E-25
1000	6.95E-12	1.44E-13	2.61E-14	6.54E-21	5.55E-18	1.74E-18	3.07E-17
1250	7.16E-08	3.23E-09	8.55E-10	4.31E-15	9.87E-13	3.76E-13	3.73E-12

Table S2: Unimolecular reaction rate constants k for the second reaction catechol $\rightarrow$ *o*-benzoquinone + H_2 as a function of temperature T and calculation method. k is given in s^{-1}, T is given in K.

T	PBE	SCAN	B3LYP	HF	MP2	DLPNO-CCSD	DLPNO-CCSD(T)
0	0.00E+00	0.00E+00	0.00E+00	0.00E+00	0.00E+00	0.00E+00	0.00E+00
273	7.57E-42	8.24E-47	2.26E-49	2.86E-76	6.77E-55	1.53E-61	8.11E-57
298	2.73E-37	7.76E-42	2.95E-44	7.98E-69	3.04E-49	2.49E-55	5.28E-51
500	2.95E-17	5.76E-20	2.30E-21	4.74E-36	2.22E-24	5.22E-28	1.98E-25
750	2.39E-07	3.74E-09	4.37E-10	7.07E-20	4.56E-12	1.74E-14	9.10E-13
1000	2.47E-02	1.09E-03	2.07E-04	9.39E-12	6.76E-06	1.04E-07	2.02E-06
1250	2.54E+01	2.09E+00	5.77E-01	7.70E-07	3.73E-02	1.32E-03	1.42E-02
